# Immune cells inhibit the tumor metastasis in the 4D cellular lung model by reducing the number of live circulating tumor cells

**DOI:** 10.1038/s41598-018-34983-7

**Published:** 2018-11-08

**Authors:** Dhruva K. Mishra, Humberto J. Rocha, Ross Miller, Min P. Kim

**Affiliations:** 1 Department of Surgery, Houston Methodist Research Institute, Houston, TX USA; 2 Department of Pathology and Genomic Medicine, Houston Methodist Hospital, Houston, TX USA; 3 Division of Thoracic Surgery, Department of Surgery, Weill Cornell Medical College, Houston Methodist Hospital, Houston, TX USA

## Abstract

The immune system and tumor microenvironment play a decisive role in tumor progression. We developed a novel model to better understand tumor progression and interaction with immune cells and the cellular components. We grew 393 P non-metastatic and 344SQ metastatic murine cells in an acellular metastatic lung cancer model, where both cell lines formed circulating tumor cells (CTC) and metastatic lesions. When the CTC from this model were placed in the tail vein of nu/nu mice, both cell lines formed metastatic lesions. However, in syngeneic immune-competent mice, the CTC from the non-metastatic cell line did not metastasize while the CTC from the metastatic cell line metastasized. When we placed the activated immune cells in the cellular lung model, it decreased CTC and metastatic lesion formation for the non-metastatic cell line while it had no impact on metastatic cell line. The metastatic cell line had a significant increase in expression of programmed death-ligand 1 (PDL-1) compared to the non-metastatic cell line in the model. Overall, the immune cells showed an impact on viability of CTC for cell lines with a decreased expression of PDL-1 that leads to decreased metastatic lesion formation. Further studies are needed to understand the subtype of immune cells and mechanism of decreased CTC viability and metastasis inhibition.

## Introduction

Tumor cells grow by developing multiple immunosuppressive mechanisms to prevent immune cells from killing the abnormal cells. Eventually, these tumor cells form metastasis, and often lead to patient mortality. Metastasis is the process by which tumor cells break the cell-cell and cell-matrix interaction in the primary tumor nodule, go through the basement membrane, travel in the circulation to a distant site as a single cell, go past another basement membrane and grow in a distant site. Recently, several immune checkpoint inhibitors such as Programmed death-1 (PD-1) and programmed death-ligand 1 (PD-L1) have been shown to improve survival rates for patients with metastatic disease^1,2^. PD-1 acts to restrain T-cell activity in inflammation, infection or cancer to limit autoimmunity and PD-1-deficient mice have shown signs of autoimmunity such as lupus-like syndrome, type I diabetes, dilated cardiomyopathy and hydronephrosis^3^. PD-1 interactions with ligand PD-L1, which leads to the inhibition of T lymphocyte proliferation, induce apoptosis of tumor specific T-cells and develop tumor cells to cytotoxic T lymphocyte attack^4–6^. Many types of human cancer cells are known to express PD-L1 including melanoma, lung cancer, breast cancer, ovarian cancer, pancreatic cancer, esophageal adenocarcinoma, kidney tumor and bladder cancer^7–11^. Anti-PD-1 antibody has been used in patients with metastatic melanoma, renal cell carcinoma and non-small cell lung cancer and is responsive in 20–25% of patients with 14% of patients having immune-related toxicities^2^. This shows that the immune system plays an important role in modulating tumor growth and metastasis and that careful regulation of the pathway can improve cancer patient survival rates. A recent animal model of metastasis shows that metastasis is regulated by the microRNA-200/ZEB1 axis that controls tumor cell PD-L1 expression and intratumoral immunosuppression^12^. However, even though there is a very promising response from inhibiting the checkpoint for T-cell activity, the response is not universal with only a small percentage of patients developing a durable response from this treatment.

We postulate that we can create a novel metastasis model that may elucidate mechanisms for immune cell driven inhibition of tumor growth and metastasis. Recently, we have developed a 4D lung cancer model that mimics the metastatic process and allows us to isolate tumor cells at different phases of tumor progression, namely at the primary tumor site, circulating tumor cells (CTC), and metastatic lesions^13–15^. Using this model, we discovered the mechanism of immune cell interaction with tumor cells and its impact on metastasis. Our study shows that immune cells decrease the CTC viability that leads to decreased metastasis for certain cell lines.

## Results

### Both murine cell lines formed metastatic lesions on the acellular *ex vivo* lung model in the absence of cellular components and immune cells

**Figure 1:**
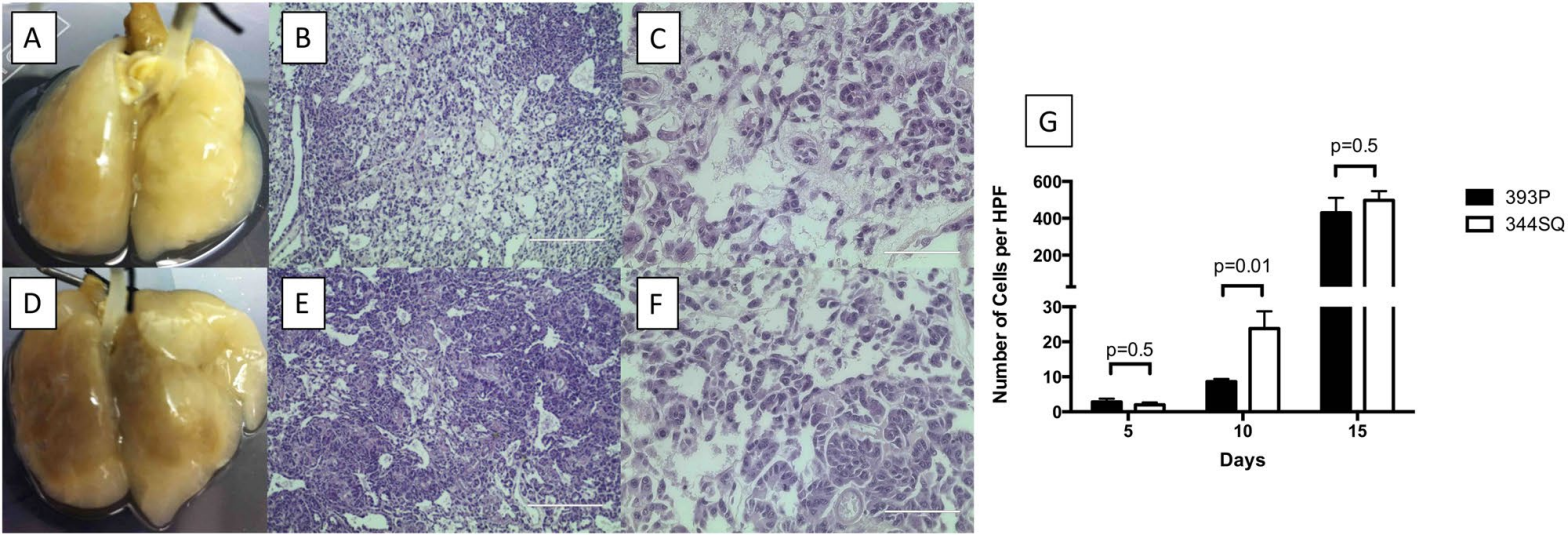
Murine cells (393p and 344SQ) formed metastatic lesions in the acellular 4D lung model irrespective to their differential nature of metastasis. (**A**–**C**) Non-metastatic 393 P cell line. Image of non-metastatic 3939 P cells grown on acellular 4D model (**A**), H&E of primary tumor (10×, B) and H&E of metastatic lesion (40×, **C**) formation by day 15. (**D**–**F**) Metastatic 344SQ cell line. Image of metastatic 344SQ cells grown on the acellular 4D model (**D**), H&E of the primary tumor (10×, **E**) and H&E of the metastatic lesion (40×, **F**). (**G**) Graph shows no significant difference in metastatic lesion formation in high power field (HPF) between 393P and 344SQ cells on acellular 4D model on day 15 (p = 0.5).

Unlike the observations we made *in vivo*, in which 344SQ cells form metastatic lesions and 393 P cells do not form metastatic lesions, when we placed either 344SQ or 393 P on the *ex vivo* acellular 4D metastasis model, where the normal cells are removed in the lung which allows observation of the behavior of cancer cells with the native matrix, both cell lines formed metastatic lesions. Briefly, *ex vivo* acellular 4D model was created by harvesting heart-lung block from Sprague dawley rat and removing native lung cells that leaves behind native extracellular matrix. The native matrix components provide intact structure of vasculature, bronchi and alveoli. The acellular lung was made into a metastasis model by tying the right main bronchus. The acellular lung is placed in a bioreactor and tumor cells (344SQ or 393 P) were placed in the the trachea which travels to the left lung and form primary tumor. The CTC forms from the primary tumor, intravasate into the vasculature, travel to the contralateral lung, extravasate and form metastatic lesions^15^. For both cell types, we observed the perfusable tumor nodule on the primary tumor side on the *ex vivo* model on day 2, which grew over time (Fig. 1). There was no difference in the primary tumor’s size by day 15 between the lung model seeded with 393 P (Fig. 1B) or 344SQ (Fig. 1E, p = 0.7). The histopathology of these matrixes on day 15 shows extensive cancer growth with multiple tumor nodules in the entire matrix covering the alveoli and bronchus except the vasculature (Fig. 1B,C,E,F). H&E staining of the primary tumor formed by 393 P (Fig. 1B) and 3344SQ (Fig. 1E) shows no difference in morphology, however, we observed tumor cell-tumor cell and cell-matrix interactions with a lack of cells in the side of the vasculature. We found live CTC with both 344SQ and 393 P cells growing on our *ex vivo* 4D model starting from day 5 after tumor cell seeding. These CTCs were obtained by collecting cell culture media that circulated in lung for 24 hours in the bioreactor through the vasculature of *ex vivo* 4D lung model, spinning them at 500 g for 5 minutes, discarding supernatant and reconstituting the pellet with 1 ml of fresh media. The percentage of live cells varies from 2% to 30% with the tumor growth and the kind of cell (393 P or 344SQ). The 344SQ tumor had more CTC up to day 7 (p = 0.0004) but the difference diminished by day 15 (p = 0.7) as compared to the 393 P cells. We measured the number of metastatic lesions per high power field at different time points. There was no difference in the number of metastatic lesions between 393 P and 344SQ on day 5 (p = 0.5). However, there were more metastatic lesions with the acellular lung model seeded with 344SQ cells compared to 393 P cells (p = 0.01) on day 10 that disappeared by day 15 (p = 0.5, Fig. 1G). In the absence of cellular components and an immune system, both cell lines formed metastatic tumor.

### Both 2D cells placed in tail veins of immunocompetent mice formed metastatic lesions while only CTC from the 4D model seeded with 344SQ formed metastatic lesions

We injected 393 P or 344SQ cells from the petri dish (2D) in the tail vein of immunocompetent mice (129 Sv) and determined the survival and pattern of nodule formation in the lungs morphologically and histologically. On the day of necropsy, the mice were either found dead or stressed because of tumor burden as per the veterinary technician. The survival analysis of 2D cells from 393 P or 344SQ cells shows a significantly worse survival with 344SQ cells (p = 0.002). The average survival time for mice with 344SQ 2D cells was 11.2 ± 2.7 days while it was 39 ± 9.1 days with 393 P 2D cells. Like the tumor cells placed in the flank of mice, both 2D cells placed in the tail vein formed metastatic lesions.

**Figure 2:**
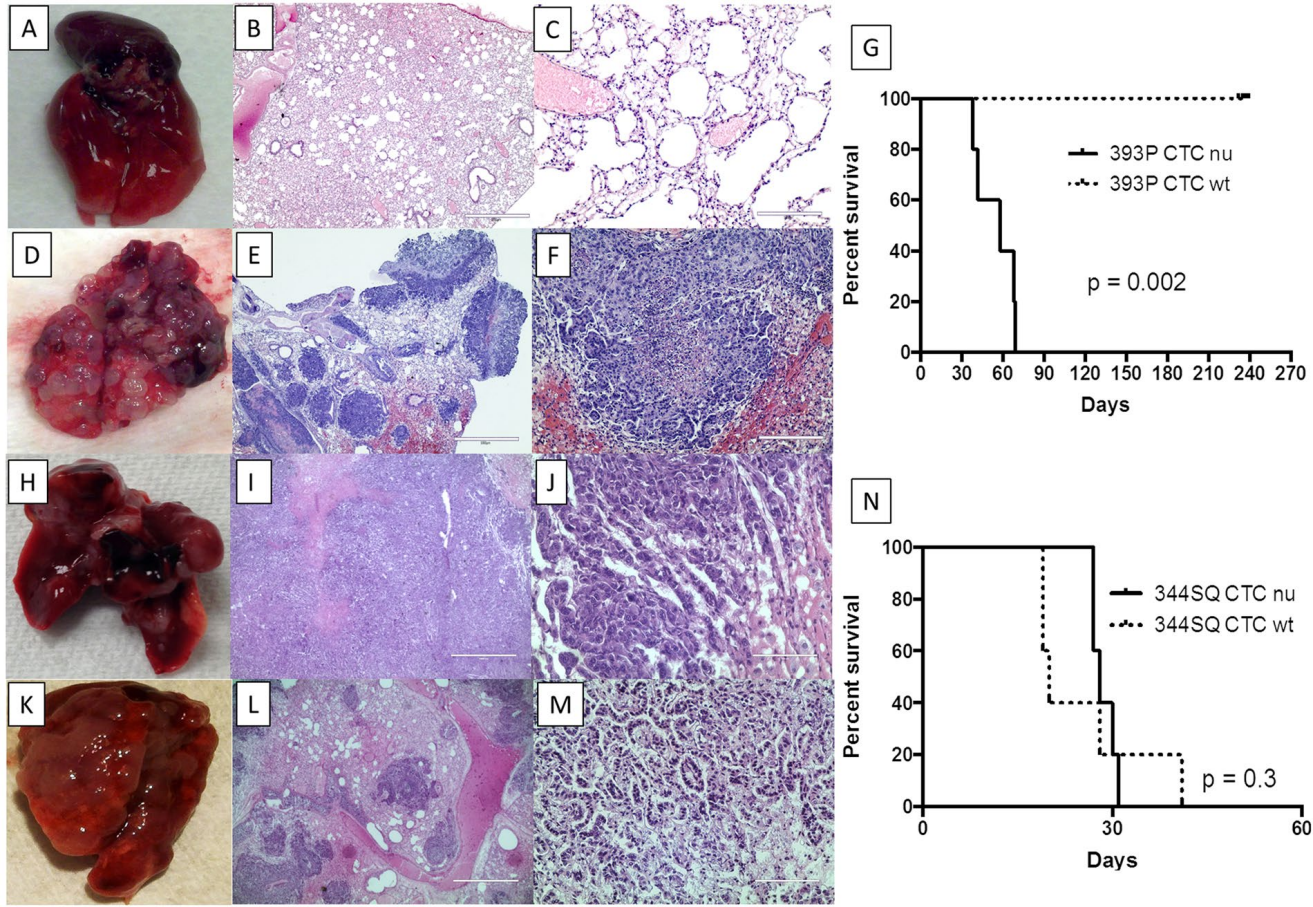
CTC from the acellular 4D model showed the differential phenotype of metastasis upon tail vein injection in immunocompetant 129 Sv mice and immunocompromised nu/nu mice from 4D model. (**A**–**C**) 393 P CTC in immunocompetent mice. Image of immunocompetent 129 Sv mice’s lung after 8 months after tail vein injection of CTC from 4D acellular model seeded with 393 P (**A**). H&E staining of the lungs with absence of any micro metastasis in low power (**B**, 10X) and high power (**C**, 40X). (**D**–**F**) 344SQ CTC in immunocompetent mice. Image of immunocompetent 129 Sv mice’s lung at necropsy after tail vein injection of CTC from the 4D acellular model seeded with 344SQ (**D**). H&E staining of the lung with metastasis with low power (**E**, 10X) and high power (**F**, 40X). (**H**–**J**) 393 P CTC in nu/nu mice. Image of nu/nu mice’s lung at necropsy after tail vein injection of CTC from 4D acellular model seeded with 393 P (**H**). (**H**,**E**) staining of the lungs with tumor at low power (I, 10X) and high power (**J**, 40X). (**K**–**M)** 344SQ CTC in nu/nu mice. Image of nu/nu mice’s lung at necropsy with gross tumor after tail vein injection of CTC from 4D acellular model seeded with 344SQ (**K**). (**H**,**E**) staining of the lung with tumor at low power (**L**, 10X) and high power (**M**, 40X). None of the immunocompetant 129 Sv mice died of cancer throughout the study and there was significantly longer survival for 129sv mice compared to nu/nu mice injected with CTC from the 4D model seeded with 393 P (**G**, p = 0.002). There was no significant difference in survival of nu/nu mice and 129 Sv mice when injected with CTC from the 4D model seeded with 344SQ (N, p = 0.3).

In order to recapture the metastatic phenomenon seen in immunocompetent mice with these two cell lines, we placed cells in the acellular lung model and isolated the tumor cells in the CTC phase to tumor progression and placed them in the immunocompetent mice. The immunocompetent mice injected with CTC from 393 P grown on the acellular 4D model survived until the end of experiment (approximately 8 months) without any signs of metastatic tumors at the time of necropsy (Fig. 2G). We found healthy and intact lungs after 8 months from the mice injected with 393 P CTC (Fig. 2A–C). There were no signs of tumors anywhere in the mice. Histological analysis of the lung shows intact and healthy bronchus, alveoli and vasculature (Fig. 2B,C). However, the CTC from the acellular 4D model seeded with 344SQ formed metastatic tumors. The mice injected with 344SQ CTC also showed large, numerous tumor nodules on the lung surface (Fig. 2D). Histological analysis of these lung tumors showed a bud-like tumor nodule with slightly different proliferation on the edges. It resembled the infiltration stage (Fig. 2E,F). This different tumor formation shows that the acellular 4D model can isolate CTC found in the mice with tumors in the flank that inhibit metastatic lesion formation for 393 P cells but not 344SQ cells. This result suggests that there is a difference in the behavior of tumor cells in different phases of tumor development. The cells from the petri dish (2D cells) are in the phase of tumor development that can grow in the lung when the tumor cells are placed in the tail vein while CTC from the acellular model are in the phase of tumor development that is similar to the CTC *in vivo* where only the metastatic tumor cells form metastasis and the non-metastatic tumor cells do not form metastasis. The presence of both the cellular component and/or immune system in the immune competent mice may play a role in the different behavior of the two cell lines.

### CTC from the 4D model seeded with either cells form metastatic lesions in nu/nu mice

In order to determine whether the immune system is necessary to form metastatic tumors, we placed CTC from the model in the tail vein of nu/nu mice. We postulated that if CTC from the 4D model seeded with 393 P or 344SQ re-capture the phenomenon seen *in vivo* in nu/nu mice, the immune system would not play an important role in metastatic lesion formation. We tested this hypothesis by injecting CTC in the tail vein of immune suppressed nu/nu mice. We found that CTC from the 4D model seeded with either 393 P (Fig. 2H–J) or 344SQ (Fig. 2K–M) formed metastatic tumors. Since both cells formed metastatic lesions in the nu/nu mice while there was a differential metastatic lesion formation in immune competent mice, we concluded that immune cells play an important role in inhibiting CTC from the 4D model seeded with 393 P from forming metastatic lesions. When CTC from the 4D model seeded with 344SQ were injected into the tail vein of nu/nu mice or wild type mice, there were no differences in the survival rates between the two types of mice (p = 0.3; Fig. 2N). However, nu/nu mice injected with CTC from the 4D model seeded with 393 P were less likely to survive compared to the wild type mice (p = 0.002, Fig. 2G).

### Activated immune cells inhibits CTC formation of 393P cells on the *ex vivo* 4D cellular lung model

**Figure 3:**
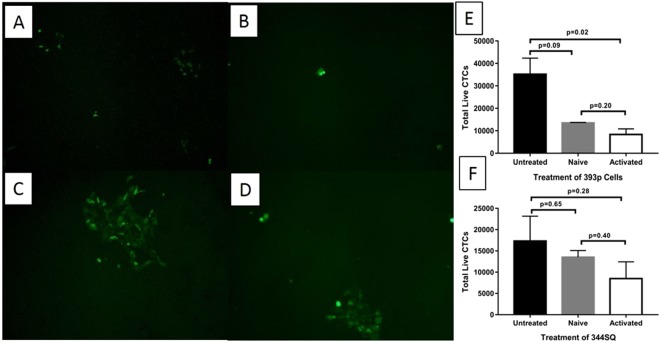
Effect of immune cells on CTC and tumor metastasis from the cellular *ex vivo* 4D lung seeded with 393p and 344SQ cells. (**A**–**D**) Immunofluorescence images (10X) of CTC. Significantly more viable GFP positive CTC appeared after 48 hours of plating CTC from the cellular lung seeded with 393 P without immune cells (**A**) compared to CTC from the cellular lung seeded with 393 P with activated immune cells (**B**). There is no difference in the number of viable GFP positive CTC from the cellular lung seeded with 344SQ without immune cells (**C**) compared to CTC from the cellular lung seeded with 344SQ with activated immune cells (**D**). (**E**,**F**) Total number of GFP positive CTC. There is significantly fewer CTC in the presence of activated immune cells in the cellular lung model seeded with 393 P cells (**E**, p = 0.02) while there is no difference with naïve immune cells (**E**, p = 0.09). There is no difference in the total number of live CTC in the presence of activated (p = 0.28) or naïve (p = 0.65) immune cells in the cellular lung model seeded with 344SQ).

Next, we wanted to determine if we could recapture the *in vivo* phenomenon on the *ex vivo* 4D cellular model. We used the cellular 4D model^14^ where all of the cells in the lung are preserved to evaluate the impact of adding immune cells in the formation of metastatic lesions. We tested naïve and activated immune cells in the model. We treated the *ex vivo* 4D cellular lung model seeded with either 393 P or 344SQ with naïve immune cells (Fig. 3E,F). There was no significant difference in the number of CTC formed in the 4D cellular model seeded with 393 P cells with naïve immune cells or without any immune cells (p = 0.09, Fig. 3E). In addition, there was no significant difference in CTC formation in the 4D cellular model seeded with 344SQ cells with or without naïve immune cells (p = 0.65, Fig. 3F). There was no inhibition of metastatic lesion formation for either group of cells.

**Figure 4:**
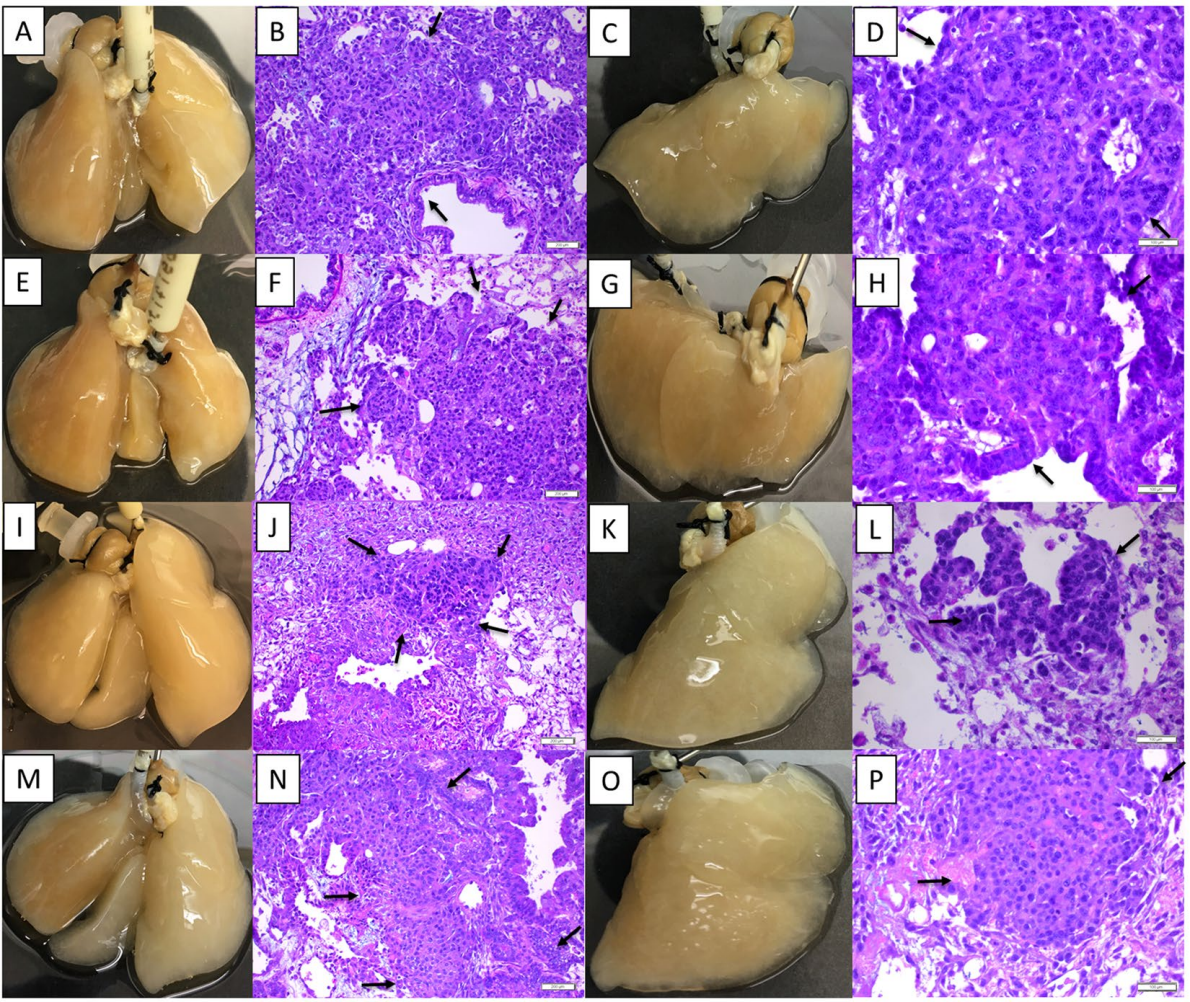
Both murine cells formed a primary tumor, CTC and metastatic lesions on the 4D cellular lung model and activated immune cells inhibit metastatic lesions formation on the 4D lung model by 393 P cells. (**A**–**D**) 393 P on 4D cellular lung model. Image of the cellular lung model seeded with 393 P cells (**A**) and H&E of the primary tumor at low power on day 7 (**B**). Image of the right lung or site of metastatic lesion (**C**) and H&E at high power (**D**) on day 14. (**E**–**H**) 344SQ on 4D cellular lung model. Image of the cellular lung model seeded with 344SQ cells (**E**) and H&E of the primary tumor at lower power on day 7 (**F**). Image of the right lung or site of metastatic lesions (**G**) and H&E of metastatic lesion at high power on day 14 (**H**). (**I**–**L**) 393 P on 4D cellular lung model treated with activated immune cells. Image of the cellular lung model seeded with 393 P cells treated with activated immune cells (**I**) and H&E of the primary tumor on day 7 (**J**). Image of the right lung or site of metastatic lesion (**K**) and H&E of the metastatic lesion on day 14 at high power (**L**). (**M**–**P**) 344SQ on 4D cellular lung model treated with activated immune cells. Image of the cellular lung model seeded with 344SQ cells treated with activated immune cells (**M**) and H&E of the primary tumor on day 7 (**N**). Image of the right lung or site of metastatic lesion (**O**) and H&E of the metastatic lesion on day 14 at high power (**P**). The arrow marks the tumor nodules.

Next, we placed the tumor cells in the *ex vivo* 4D cellular lung model to determine the impact of adding activated immune cells to the cellular model in tumor progression for 393 P and 344SQ cells. The primary tumor was well formed on the cellular lung model by both 393 P (Fig. 4A,B) and 344SQ (Fig. 4E,F) cells with peribronchiolar distribution and there was no significant effect on primary tumor growth, though tumor size was smaller compared to the acellular model. Both mouse tumor cells (Fig. 4B,F) formed solitary nodules in the rat lung. The lung matrix was intact in untreated as well as activated immune cells treated cellular models and the tumor histology was more like non-small cell lung cancer (Fig. 4B,F,J,N). Mononuclear cells were randomly distributed in primary tumor tissues (Fig. 4J,N). The activated immune cell treatment significantly reduced the amount of CTC in circulation from 393 P grown lung models (35172 ± 8276 vs 10592 ± 4284; p = 0.02, Fig. 3A,B,E) while there was no significant difference in the number of CTC for the 344SQ primary tumor with or without activated immune cells (17317 ± 10124 vs 8485 ± 6819; p = 0.28, Fig. 3C,D,F). Furthermore, the activated immune cell treatment significantly inhibited the metastatic lesion in the 393 P grown cellular model (p = 0.008), while there was no significant impact on the metastatic lesion formation in 344SQ grown model (p = 0.345). Thus, activated immune cells placed in the 4D cellular model inhibit the formation of metastatic lesions for 393 P cells but do not inhibit metastatic lesions in the 344SQ model.

### PDL1 gene is overexpressed in primary tumor of metastatic cell line

**Figure 5:**
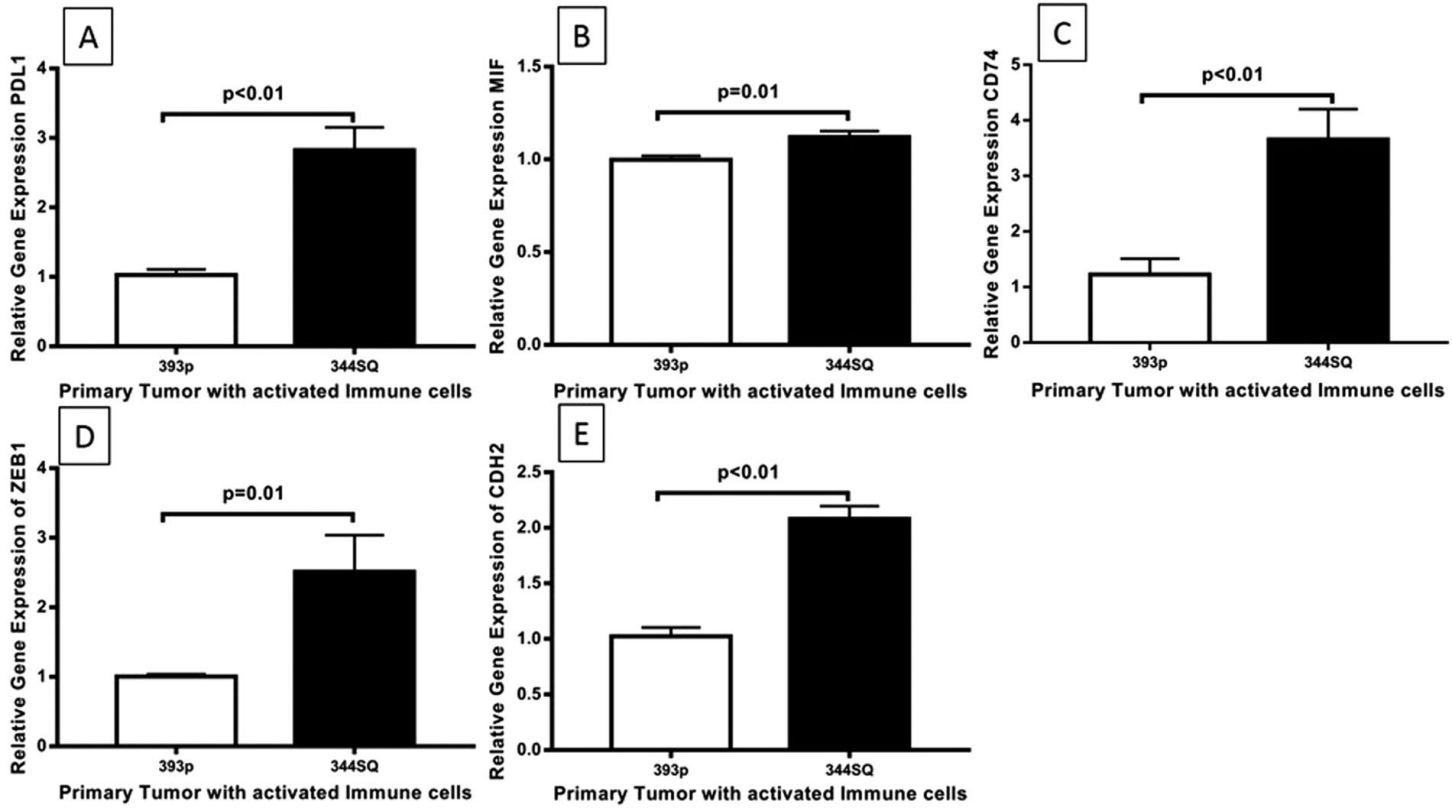
Differential expression of selected genes showed upregulation of genes associated with immune modulation and metastasis in 344SQ cells on cellular 4D lung model treated with activated immune cells. Differential Gene Expression pattern of PDL1 (**A**), MIF (**B**), CD74 (**C**), ZEB1 (**D**) and CDH2 (**F**) in primary tumors derived from 393 P and 344SQ cells, treated with activated immune cells. Genes associated with immune modulation (**A**–**C**) and mesenchymal characteristics (**D**,**E**) are significantly upregulated in 344SQ cells as compared to 393 P cells, which showed inhibition of metastasis on the 4D lung model upon activated immune cell treatment.

We compared the genes associated with immune modulation and metastasis between 393 P and 344SQ cells treated with activated immune cells on the *ex vivo* 4D lung model. Our results showed significant upregulation of PDL1 (2.8 fold, p < 0.01, Fig. 5A), MIF (1.12 fold, p = 0.01, Fig. 5B) and CD74 (3.6 fold, p < 0.01, Fig. 5C) genes in the 344SQ-lymphocyted treated model, which modulate the immune responses. It also showed the upregulation of mesenchymal genes such as ZEB1 (2.5 fold, p = 0.01, Fig. 5D), CDH2 (2.0 fold, p < 0.01, Fig. 5E) in 344SQ cells on the *ex vivo* 4D lung model. Furthermore, 344SQ cells showed significant upregulation of PDL1, CD74, ZEB1 and CDH2 (p < 0.05, Supplementary Figure 1) as compared to 393 P cells when grown on a petri dish (2D) cell culture.

## Discussion

Recently, cancer immunotherapy has attracted significant attention for various cancer treatments by boosting the body’s natural defense actively or passively. Immune responses, both innate (macrophages, granulocytes, mast cells, NK cells and DCs) and adaptive (B-cells, CD8^+^ and CD4^+^) play a crucial role in cancer metastasis by inhibiting the growth of tumors, in particular, by responding to disseminated disease. Our understanding of the role of immune cells in antitumor response has grown over the past decade, but studies to better understand cancer immunotherapy by examining the interaction between tumor cells and immune cells in the presence of a natural microenvironment have been limited due to the lack of a model that can mimic the biological process. In this study, we have successfully presented an *ex vivo* 4D lung model that mimics lung cancer growth and metastasis along with immune cell interaction and its inhibitory effect on tumor metastasis.

Our results show that activated immune cells play an important role in the inhibition of metastasis in *ex vivo* and *in vivo* models by affecting the viability of CTC. Both metastatic and non-metastatic cell lines formed the primary tumor on the *ex vivo* 4D lung model and *in vivo* upon subcutaneous injection. Gibbon *et al*.^16^ injected these syngeneic cell lines subcutaneously in immunocompetent, syngeneic mice and found that both formed the subcutaneous tumor, but only 344SQ developed metastatic disease. In the *ex vivo* acellular 4D lung model, both cell lines developed metastasis in the absence of cellular components of the tumor microenvironment and immune cells. The presence of a natural matrix allowed both cell types to form CTC and metastatic lesions. This experiment highlights the importance of the tumor microenvironment in tumor progression.

In order to determine if the difference in the results are due to the cellular component or immune cells, we injected CTC from the model into an immunocompetent mouse. In the immunocompetent mice, the non-metastatic cell line did not form metastasis while metastatic cell line formed metastasis. As a control, we injected 2D cells in the tail vein and as seen with the subcutaneous injection, the injection of the cells led to the formation of a tumor in the lung. Thus, the CTC from the model mimic the behavior of CTC seen *in vivo* when a tumor is injected in the flank. In order to delineate whether the cellular component and/or immune cells are needed to create different metastatic lesion formation by two different cell types, we injected CTC from the model in nu/nu mice. When CTC from the model seeded with either metastatic or non-metastatic cell lines were placed in the nu/nu mice, both tumor cells formed metastatic lesions. This highlights the importance of immune cells to inhibit metastatic lesion formation for non-metastatic cell lines. Our study suggests that the CTC’s interaction with immune cells plays an important role in differential metastatic behavior. The clinical relevance of CTC in the metastasis and prognosis is well established^17,18^ but the difference in the ability for some tumors to form metastatic lesions while others are killed may be due to the presence of the immune cells. If CTC are not genetically programmed to escape the immune surveillance system^19,20^, they will not survive and lose viability and ultimately metastatic potential. Our study supports the important interaction between the immune cells and CTC in the ultimate formation of metastatic lesions^21^. This difference was further highlighted when we placed the immune cells in the cellular model that showed a decrease in viable CTC and metastatic lesions for non-metastatic cell lines while there was no impact with metastatic cell lines. Our study shows that the behavioral difference may be due to the upregulation of genes associated with immune response modulation especially PD-L1 and mesenchymal characteristics in metastatic cell line that is not seen in the non-metastatic cell line.

Overall, our study showed that activated immune cells play an important role in CTC survival and ultimately the inhibition of metastasis. Further studies with a sub-population of immune cells are needed to better understand the interaction with tumor cells as well as the exact mechanism of immune cells causing apoptosis of CTC.

## Materials and Methods

### Animal handling and cell lines

The Institutional Animal Care and Use Committee (IACUC) at the Houston Methodist Research Institute approved the protocols for animal experiments. All of the animal experiments were carried out in accordance with all applicable laws, regulations, guidelines, and policies governing the use of laboratory animals in research.

We used Sprague-Dawley rats, nu/nu mice and 129 Sv mice as per approved protocol. Murine cell lines 393 P and 344SQ were kindly provided from Dr. Jonathan Kurie’s lab (MD Anderson Cancer Center, Houston, TX). Mice cell lines were derived from tumor tissues *p53*^*R172HΔg/*+^ *K-ras*^*LA1/*+^ mice^16^. 393 P tumor cells were obtained from primary lung tumors while 344SQ cells were from a subcutaneous site. These cells were cultured in RPMI1640 (Gibco, USA) with 10% FBS (Gibco, USA) and antibiotics (100 IU/mL penicillin, 100 µg/mL streptomycin, and 0.25 µg/mL amphotericin; Gibco, USA) at 37 °C in 5% CO_2_. Once the cells were 85% confluent, they were washed with PBS and subjected to trypsinization using 0.25% trypsin (Gibco, USA) to collect the cells from the flasks. The cells were washed with media and suspended in 30 to 50 mL of complete media.

### Lentiviral transduction of cells with GFP

To create the stable GFP expressing cell lines, we used the lentiviral gene expression system (Lenti-CMV-GFP-2A-Puro-Blank) from Applied Biological Materials (ABM, Richmond, BC, Canada) and transduced the murine cancer cell lines in the laboratory. The target cells (393p and 3443SQ) were plated in a 24-well plate, 24 hours prior to viral infection at a density of 0.5 × 10^5^ cells per well with 0.5 mL complete optimal medium. We replaced the growth media the next day with 0.5 mL polybrene-media-mix and added an appropriate volume (MOI = 100) of the virus to transduce cells. One well of non-transduced cells was used as an additional standard control and the plate was incubated at 37 °C with 5% CO_2_ overnight. The culture medium was replaced with 1 mL of complete medium and again incubated 37 °C with 5% CO2 overnight. The following day, the cells were split 1:3 and puromycin (Sigma-Aldrich, MO, USA) was added at the concentration of 5 µg/ml. After 7 days of selection, cells were obtained and the GFP expression was validated under a fluorescent microscope.

### Tail vein injection of tumor cells in syngeneic (129 Sv) and nu/nu mice

We used wild type immune-competent 129 Sv (Charles River, MA, USA) and nu/nu (Envigo, USA) male mice that were least 8–10 weeks old for our *in vivo* studies. These mice were divided in different groups with at least 5 mice in each group. Monolayer (2D) cells and circulatory tumor cells from each cell line (393 P and 344SQ) were injected into mice through the tail vein. Our veterinary technician assisted us with this procedure. For each type of cells, 100,000 cells were dispensed in 100 uL of complete media and carried to the animal facility on ice. Mouse restraint was used during the tail vein injection. Each group of mice was labeled by punching their ears. The mice were monitored daily by animal facility staff and weekly by us. The mice were immediately sacrificed upon signs of morbidity and necropsy was performed to find the tumors in lung, liver, kidney and elsewhere. Nodules in the lung were counted if possible and images were taken. The lungs were collected from each mouse for hematoxylin and eosin (H&E) staining to see the microscopic lesions.

### *Ex vivo* 4D acellular and cellular lung model

We used the acellular and cellular lung models for this study. We harvested the lung–heart block from 4–6 week old male Sprague-Dawley rats as described earlier^22^. We performed ventriculotomy to expose the right and left ventricles and placed a custom-made prefilled 18-gauge stainless steel needle (McMaster Carr, USA) through the right ventricle into the main pulmonary artery. This was secured with a 2–0 silk tie (Ethicon, San Angelo, TX, USA). We also placed a female luer bulkhead (Cole-Parmer, Vernon Hills, IL, USA) in the left ventricle and secured it with a 2-0 silk tie. We flushed the pulmonary artery cannula with heparinized PBS and placed it in a container containing heparinized PBS. We used this lung for the cellular model. In order to create an acellular model, we performed the decellularization process on the lung–heart block by placing TritonX and SDS as described previously^22^. Both the acellular and cellular lung models were immediately placed in a bioreactor as described below. A simplified, small, closed-system bioreactor was set up in an incubator for the lung cell culture. We used a custom-designed 500-mL glass bottle with three holes in the cap fitted with a female luer thread-style panel (Cole-Parmer), one for the pulmonary artery cannula, one for the trachea cannula, and one for the circulation of medium from the bottle. The medium was constantly circulated at 6 cc/min with the help of a Masterflex pump (Cole-Parmer, USA) through a 10-foot length of silicone oxygenator tubing wrapped around a mesh of wire solenoid (Cole-Parmer, USA). For controlled flow through the pulmonary artery, it was connected to a 3-way stopcock (Smith Medical, Dublin, OH, USA). The bottle was filled with 200 mL of complete medium that was circulated through the oxygenator tubing to prevent air bubbles.

Before seeding the murine lung cancer cells into the lung matrix, the trachea was cannulated using an 18-gauge needle, and the scaffold was fixed to the bioreactor bottle in a hanging position. To modify it for the metastasis model, we tied the right main bronchus with a silk tie that was left there for the entire experiment and placed it in the bioreactor. The murine cell lines were diluted in 50 mL of complete media and seeded into the left lung lobes through the tracheal cannula via a sterile syringe fed by gravity. After 30 minutes, we perfused the scaffold at a flow rate of 6 mL/min. The culture medium in the bottle was changed daily to make sure the nutrients were optimal for cell growth and CTC were spun down and counted. We grew the cells on the matrix for 14 days. The lung matrix was carefully removed from the bioreactor bottle, maintaining sterile conditions, and a lobectomy performed under a culture hood by tying the anatomic lobe with a 2-0 silk tie and resecting it on different days.

### Immune cell isolation and treatment of 4D model with tumor cells

To mimic the role of tumor cell specific immune cells in metastasis inhibition on the 4D lung cancer model, we collected the immune cells from mice challenged with the same tumor cells (activated immune cells). We used immune-competent wild type 129 Sv male mice that were at least 6–8 weeks old to subcutaneously inject the GFP labeled 393 P and 344SQ cells (Charles River, MA, USA). These mice were divided in 2 groups with 10 mice in each group. One million monolayer (2D) cells from each cell line (393 P and 344SQ) were injected into the flanks of mice. The mice were monitored daily by animal facility staff and weekly by us. After 10 days, the first 3 mice were sacrificed from each group and the spleen was harvested in RPMI culture media. In order to obtain naïve immune cells, we sacrificed 129 Sv mice and obtained spleen without undergoing injection of tumor cells. The spleen was transported to the lab on ice and minced using 10 mL syringe plunger. It was filtered through a 40-micron strainer and single cell suspension was pelleted. The RBCs were lysed using RBC lysis buffer (Biolegend, USA), washed with PBS and finally suspended in complete culture media. The immune cells were counted and equally divided in three aliquots (for triplicates) to treat each *ex vivo* 4D lung model with 393 P and 344SQ tumor cells. We placed them through a three-way stopcock into the pulmonary artery of the lung. We treated the 4D model with immune cells on day 0, day 3 and day 5. On day 7, the primary tumor (left lobes) was removed, however, the metastatic lobes (right lobes) were maintained until day 14.

### Flow sorting of GFP positive cells

CTC collected from the bioreactor were washed twice with PBS (with 2% FBS). The cells were suspended in complete RPMI media containing DAPI and incubated at room temperature in the dark for 10 minutes. Following incubation, the cells were spun down and finally suspended in complete RPMI media. A fraction of unstained cells were used as a negative control group. Finally, at the core facility (BD NIR Ariall, USA), GFP positive and DAPI negative cells were counted and collected in RPMI complete media.

### Gene expression analysis

The total RNA was extracted from the primary tumor using Direct-zol RNA miniPrep (Zymo Research, Irvine, CA, USA). RNA quality and quantity was determined using a Nanodrop 1000 spectrophotometer (Thermo Scientific, Waltham, MA, USA). cDNA was prepared using a high-capacity cDNA reverse transcription kit (Applied Biological Materials, Canada) with 500 ng of total RNA and qPCR assay was performed with Bright green reagent (Applied Biological Materials, Canada). We used B2M as a housekeeping gene and primers were designed for the other target genes (Table 1). A relative fold of gene expression was calculated using 2^−(ΔΔCt)^ formula.

**Table 1:** Primer sequences.

Gene	Forward (5′-3′)	Reverse (5′-3′)
PDL1	TGCGGACTACAAGCGAATCACG	CTCAGCTTCTGGATAACCCTCG
MIF	GCCAGAGGGGTTTCTGTCG	GTTCGTGCCGCTAAAAGTCA
CD74	CGCCTAGACAAGCTGACCAT	TGTTGCCGTACTTGGTAACG
ZEB1	GCCATGAGAAGAACGAGGAC	CACTTGAACTTGCGGTTTCC
CDH2	AGTTTCTGCACCAGGTTTGG	ATGTTGGGTGAAGGTGTGCT
B2M	TTCTGGTGCTTGTCTCACTGA	CAGTATGTTCGGCTTCCCATTC

### Histopathology

After lobectomy, the lung tissues were placed in 10% paraformaldehyde and shipped to the Pathology Core Laboratory at Houston Methodist Hospital for further processing. Briefly, the tissues were fixed in 10% formalin overnight, processed, and embedded in paraffin. H&E staining was performed to identify cancer regions. The embedded tissues were cut into 4-micron slides and dewaxed; antigen retrieval was performed with antigen-unmasking solution (H-3300; Vector Laboratories, Burlingame, CA, USA) in a steamer for 20 minutes. The slides were cooled for 20 minutes at room temperature, washed in PBS, and stained for H&E following standard protocols [12]. Expert board-certified pathologists examined the stained slides, and images were captured using a microscope (EVOS, Fisher Scientific, USA). The metastatic lesions were determined by coverage of tumor cells per high power field (40X) in random areas.

### Statistical analysis

All analyses were performed using PRISM Version 5.0 software (GraphPad Software, La Jolla, CA, USA). Independent two-sample t-tests were performed to compare between the groups. A p < 0.05 was considered to be statistically significant.

## Electronic supplementary material

Supplementary Figure 1

## Data Availability

No datasets were generated or analyzed during the current study.
