# Supplementary material for: Immune cells inhibit the tumor metastasis in the 4D cellular lung model by reducing the number of live circulating tumor cells
Source: Sci Rep. 2018 Nov 8;8:16569. doi: 10.1038/s41598-018-34983-7 (PMC6224572; doi:10.1038/s41598-018-34983-7)
Supplement: Supplementary file 1 — Supplementary Figure 1 [file 41598_2018_34983_MOESM1_ESM.pdf]

Immune cells inhibit the tumor metastasis in the 4D cellular lung model by reducing the number of live circulating tumor cells

Dhruva K. Mishra[1], Humberto J. Rocha[1], Ross Miller[2], Min P. Kim[1,3]

[1]Department of Surgery, Houston Methodist Hospital Research Institute, Houston, TX; [2]Department of Pathology and Genomic Medicine, Houston Methodist Hospital, Houston, TX; [3]Division of Thoracic Surgery, Department of Surgery, Weill Cornell Medical College, Houston Methodist Hospital, Houston, TX

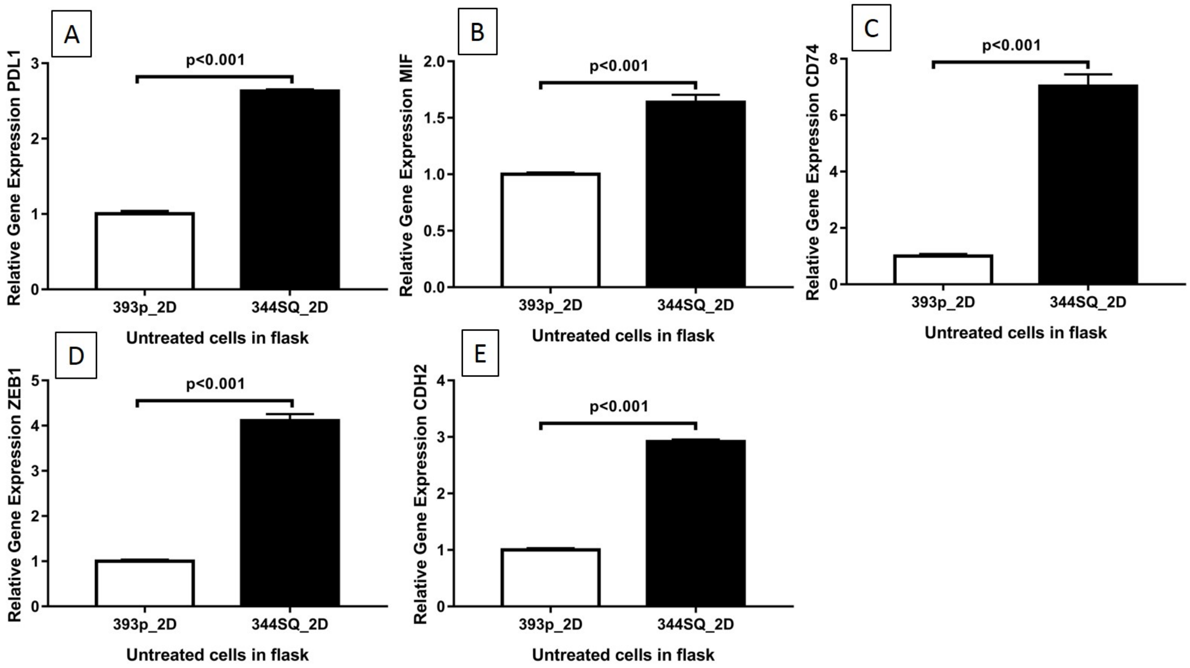

Supplementary Figure 1: Differential expression of selected genes showed upregulation of genes associated with immune modulation and metastasis in 344SQ cells in 2D flask cell culture. Differential Gene Expression pattern of PDL1 (A), MIF (B), CD74 (C), ZEB1 (D) and CDH2 (F) in 393P and 344SQ 2D cells. Genes associated with immune modulation (A-C) and mesenchymal characteristics (D-E) are significantly upregulated in 344SQ cells as compared to 393p cells.
